# Association between Int7G24A rs334354 polymorphism and cancer risk: a meta-analysis of case-control studies

**DOI:** 10.1038/srep11350

**Published:** 2015-06-15

**Authors:** Weixiang Wu, Yeqing Tong, Xiaoyun Wei, Qiang Zhao, Xiaoqi Pan, Guangxia Yu, Qing Lu

**Affiliations:** 1 Key Laboratory of Environment and Health, Ministry of Education & Ministry of Environmental Protection, and State Key Laboratory of Environmental Health (Incubating), School of Public Health, Tongji Medical College, Huazhong University of Science and Technology, P13 Hangkong Road, Wuhan, Hubei, 430030, China; 2 Hubei provincial center for disease control and prevention.

## Abstract

Accumulating evidences have suggested the potential association between Int7G24A (rs334354) polymorphism and cancer risk. However, results from epidemiological studies are controversial. We thus conducted this meta-analysis to clarify the association. Relevant studies were identified on electronic databases according to the inclusion criteria. A total of 13 case-control studies containing 4092 cases and 5909 controls were included in our meta-analysis. Odds ratios (ORs) with 95% confidence intervals (CIs) were applied to assess the association. The results of the overall population had suggested that Int7G24A polymorphism had an increased risk for cancer, reaching significant levels in the 2 genetic models (allele model, OR = 1.25, 95% CI 1.09-1.42, *P* = 0.001; dominant model, OR = 1.24, 95% CI 1.06-1.46, *P* < 0.008). Besides, significant association was found among Asian population (allele model, OR = 1.27, 95% CI 1.11-1.45, *P* < 0.001; dominant model, OR = 1.28, 95% CI 1.11-1.49, *P* < 0.001), whereas there was non-significant relationship detected among Caucasian population (allele model, OR = 1.08, 95% CI 0.92-1.26, *P* = 0.352; dominant model, OR = 1.05, 95% CI 0.87-1.26, *P* = 0.639). The present meta-analysis had suggested that Int7G24A polymorphism of gene TGFBR1 involved in the transforming growth factor beta (TGF-β) signaling pathway had a significantly increased risk for cancer development.

Cancer has become the leading cause of death in developed countries and the second leading cause of death in developing countries, according to the data provided by the International Agency for Research on Cancer (IARC)^1^,^2^. In the next decades, the burden of cancer will be heavier since the world population is increasing and the problem of ageing is getting worse^3^. A number of measures have been recommended for the cancer prevention, which have made great progress. However, the etiology of cancer still remains unclear.

In the recent years, interest in the genetic susceptibility to cancers has led to a growing attention to the study of polymorphisms of genes involved in tumourigenesis. TGF-β is one of the most potent inhibitors of proliferation in epithelial, neuronal and hematopoietic cells^4^,^5^. Several important biological events are governed by this growth factor, such as cell growth, tissue differentiation, production and degradation of extracellular matrix, morphogenesis, and apoptosis^4^,^6^. Alterations of TGF-β superfamily signaling have been implicated in various human pathologies, including cancer, developmental disorders, cardiovascular and autoimmune diseases^6^,^7^,^8^. As a key member within the TGF-β signaling way, the gene polymorphism of TGF-β receptor type I (TGFBR1) had been reported to be related with cancer risk^9^,^10^,^11^.

Among the polymorphism variations of gene TGFBR1, Int7G24A (rs334354), representing a G to A transversion in the +24 position of the donor splice site in intron 7, had been firstly found to be possibly related with cancer risk by Chen in 1999^12^. After that, several studies had reported the potential associations between Int7G24A rs334354 genotype and the risk for kidney, breast and lung cancer^13^,^14^. However, the relationship remains inconclusive, possibly due to the limited sample size. Thus, meta-analysis was applied for combining the studies with small sample size to draw a more reliable conclusion by calculating a pooled risk estimate. A meta-analysis of 3 studies conducted 9 years ago indicated that Int7G24A might be a tumor susceptibility allele for non-small cell lung cancer (NSCLC), kidney and bladder cancer^15^. Another meta-analysis performed by Zhang found non-significant association between Int7G24A rs334354 polymorphism with colorectal cancer^16^. However, there were more rigorous case-control studies on the association between Int7G24A with colorectal cancer conducted these years. Therefore, we performed an updated meta-analysis of all available case–control studies applying 5 genetic models to obtain a more reliable conclusion. Besides, subgroup analysis by ethnicity, genotyping method and cancer type were also conducted for further study. To our knowledge, this is the most comprehensive meta-analysis with the most included studies regarding the Int7G24A rs334354 polymorphisms and cancer risk.

## Results

### Characteristics of studies

**Figure 1:**
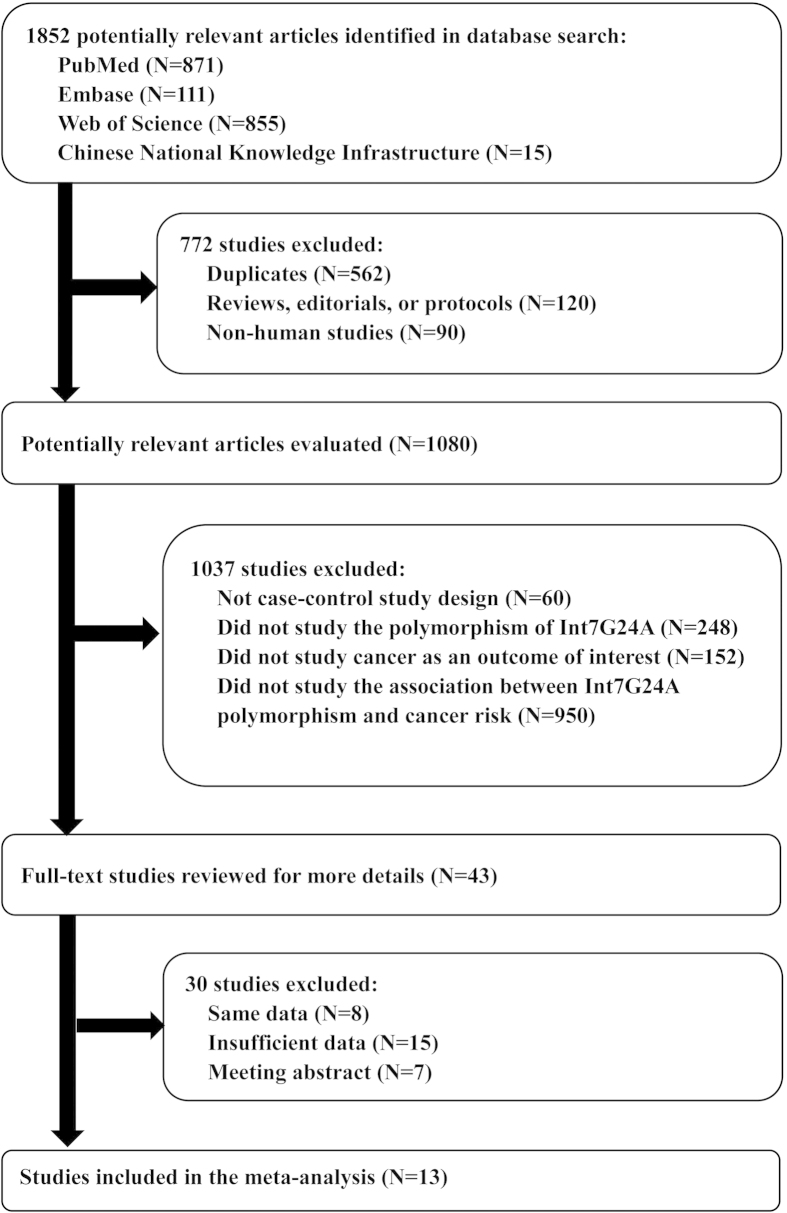
Flow chart of the study identification and selection progression. The terms “N” in the boxes represent the number of corresponding studies. The term “same data” means the studies which reported their results based on the same data. The term “insufficient data” refers to the studies which did not provide enough data for us to calculate the ORs and 95% CIs of the association between Int7G24A polymorphism and cancer risk.

In this meta-analysis, 13 studies^12^,^13^,^14^,^17^,^18^,^19^,^20^,^21^,^22^,^23^,^24^,^25^,^26^ were identified on the electronic databases (PubMed, Embase, Web of Science and Chinese National Knowledge Infrastructure) according to the inclusion and exclusion criteria. The study identification and selection progression was summarized in Fig. 1. Totally, 13 studies containing 4092 cases and 5909 controls were included in our meta-analysis and their main characteristics were shown in Table 1. These studies included 4 colorectal studies, 2 breast cancer studies, 1 acute lymphocytic leukemia studies (including T-lineage and B-lineage), 1 cervix cancer study, 1 non-small cell lung cancer study (NSCLC), 1 osteosarcoma study, 1 renal cell carcinoma (RCC) and transitional cell carcinoma study of upper urinary tract and bladder (TCC) study, 1 esophageal squamous cell carcinoma (ESCC) study, and 1gastric cardia adenocarcinoma (GCA) study. Among the 13 studies, 5 studies were performed in China, 1 in German, 3 in Sweden, 2 in USA, 1 in Netherlands and 1 in Spain. Of these studies, there were 5 studies of Asian, 5 studies of Caucasian and 3 studies of mixed ethnicity (both of them were the mixed population of Caucasian and African-American). As for the control source, 5 studies applied population-based (PB) control, while the other 8 studies performed their studies employing hospital-based (HB) control. In addition, genotyping methods were various between studies, among which 6 studies applied polymerase chain reaction-restriction fragment length polymorphism (PCR-RFLP), 4 studies used polymerase chain reaction-single strand conformation polymerase (PCR-SSCP) and 3 studies employed TaqMan. The genotype distributions in the controls of all studies were in agreement with Hardy–Weinberg equilibrium (HWE) except for 1 study^24^. The estimated quality of all included studies was in the range of 3-9 scores. The ratings had been reported in Table 1.

### Association between Int7G24A rs334354 polymorphism and cancer risk

**Figure 2:**
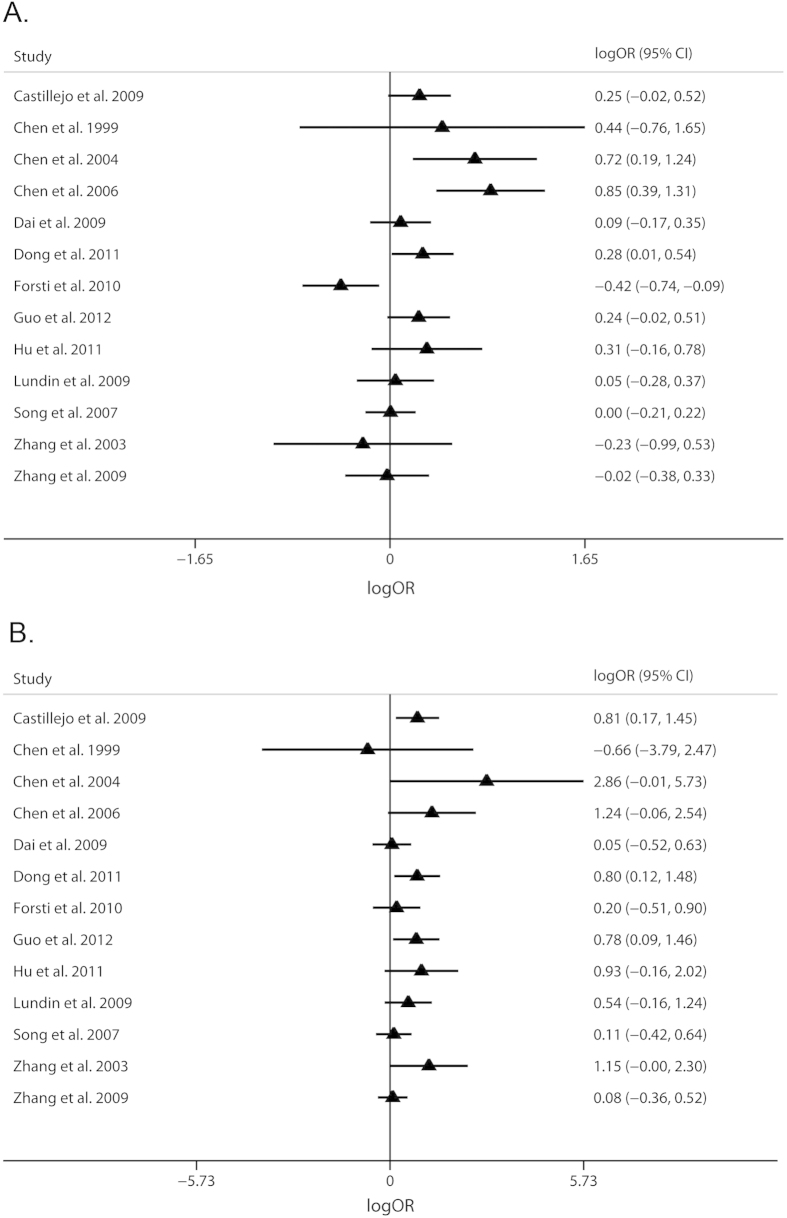
Plots of the study-specific estimates and 95% CIs of the two parameters , **A**) for *θ*_2_; **B**) for *θ*_3_. *θ*_2_ is the logarithmic scale of OR_GA/GG_ and *θ*_3_ is the logarithmic scale of OR_AA/GG_ derived from logistic regression. The triangles and horizontal lines correspond to the study-specific estimates and 95% CIs.

**Figure 3:**
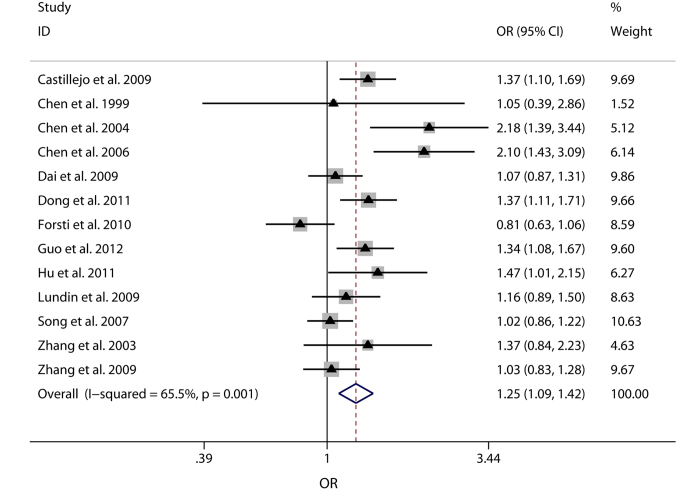
Forest plots of OR with 95% CI for Int7G24A rs334354 polymorphism and cancer risk applying allele model (A vs. G). The triangles and horizontal lines correspond to the study-specific ORs and 95% CIs. The gray areas reflect the study-specific weight. The hollow diamonds represent the pooled OR and 95% CI of the overall population. The vertical solid lines show the OR of 1 and the vertical dashed lines indicate the corresponding pooled ORs.

**Figure 4:**
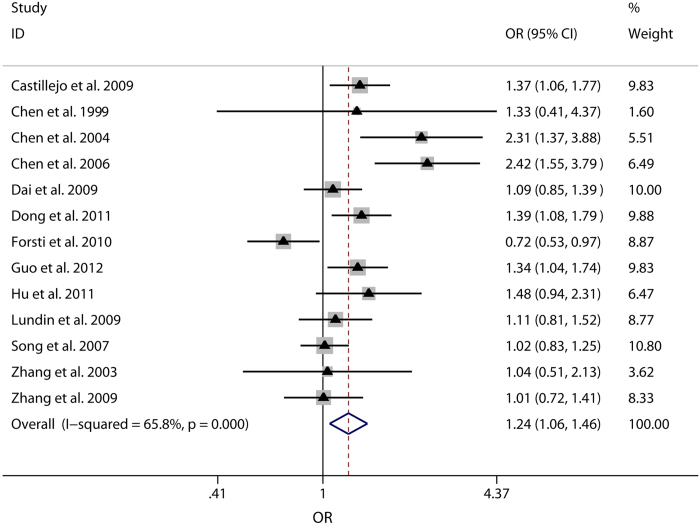
Forest plots of OR with 95% CI for Int7G24A rs334354 polymorphism and cancer risk applying dominant model (GA + AA vs. GG). The triangles and horizontal lines correspond to the study-specific ORs and 95% CIs. The gray areas reflect the study-specific weight. The hollow diamonds represent the pooled OR and 95% CI of the overall population. The vertical solid lines show the OR of 1 and the vertical dashed lines indicate the corresponding pooled OR.

In our meta-analysis, the Q test of OR_GA/GG_ and OR _AA/GG_ were *P*_h_ < 0.001 and *P*_h_ = 0.168. Thus, the random-effect model was applied to calculate the summary ORs. Under logistic regression, the OR_GA/GG_ and OR _AA/GG_ were 1.19 (95% CI 1.03-1.38, P = 0.021) and 1.80 (95% CI 1.30-2.49, P < 0.001). The parameters *θ*_2_ and *θ*_3_ in the logistic regression equation were 0.08 (95% CI 0.01-0.14) and 0.26 (95% CI 0.11-0.40). In addition, plots of study-specific estimates and 95% CIs of the two parameters, *θ*_2_ and *θ*_3_, had been shown in Fig. 2. According to our results, the *P* value of 0.711 for the null hypothesis had suggested there was no difference between OR_GA/GG_ and OR _AA/GG_. Therefore, the best genetic model was indicated to be dominant model according to the above algorithm. Besides, in order to explore whether A allele could increase the risk for cancer or not, allele model (A vs. G) would also be conducted. Forest plots of meta-analysis on the association between Int7G24A rs334354 polymorphism and cancer risk applying the 2 models were displayed in Figs 3 and 4. The overall effects of Int7G24A rs334354 mutation on the risk for cancer were investigated in 13 studies with 4092 cases and 5909 controls. When the dominant model was applied, significantly increased risk was found with OR of 1.24 (95% CI 1.06-1.46, *P* = 0.008). As for the allele model, increased risk was determined with OR of 1.25 (95% CI 1.09-1.42), reaching a significant level (P = 0.001). However, heterogeneity were confirmed in both of the two models (*P*_h_ < 0.001 for dominant model, *P*_h_ = 0.001 for allele model).

### Subgroup analyses

**Figure 5:**
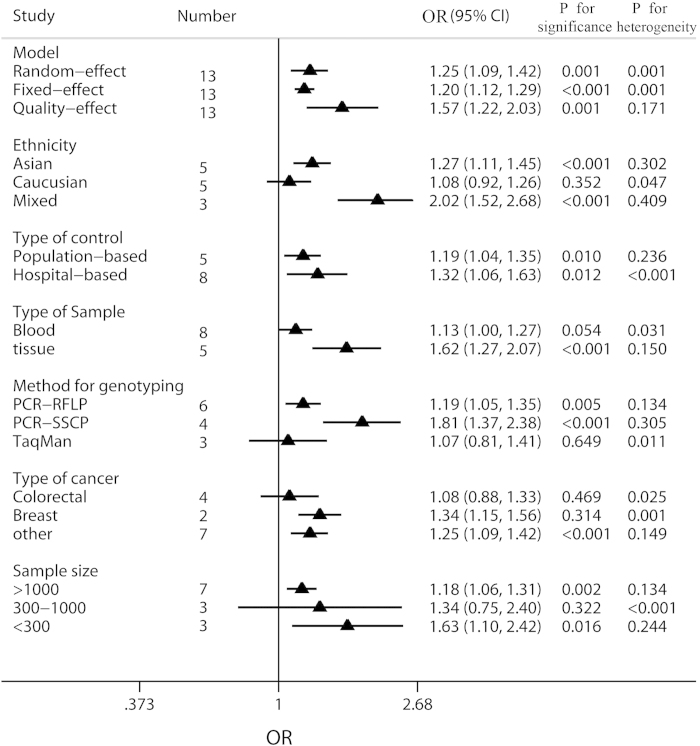
Meta-analysis of the association between Int7G24A polymorphism and cancer risk applying allele model (A vs. G). The triangles and horizontal lines correspond to the subgroup-specific ORs and 95% CIs. The vertical solid line shows the OR of 1. Especially, “Other” indicates other kind of cancer in addition to breast cancer and colorectal cancer, including 2 ALL studies (T-lineage and B-lineage), 1 cervix cancer study, 1 NSCLC study, 1 osteosarcoma study, 1 RCC study, 1 TCC study, 1 ESCC study, 1 colon adenoma study, and 1 GCA study; “Mixed” means mixed population with Caucasian and African-American.

**Figure 6:**
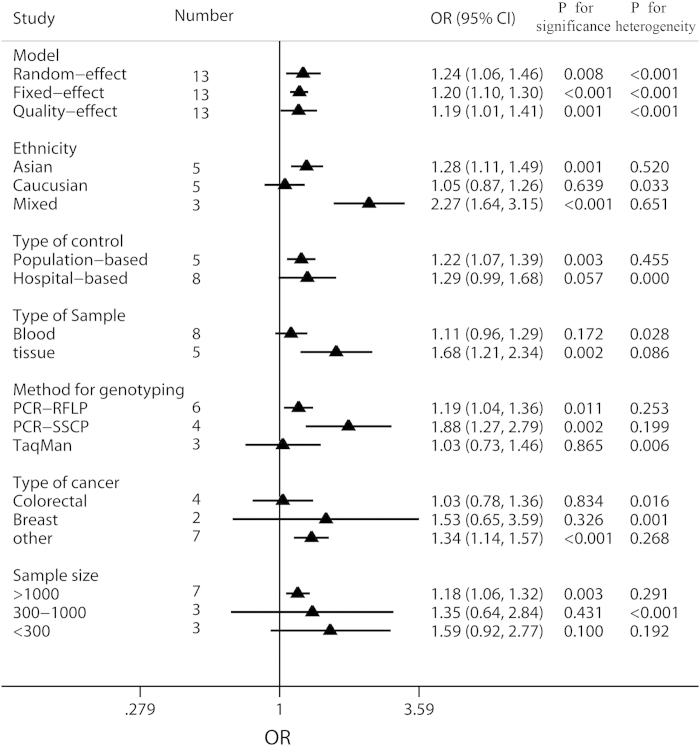
Meta-analysis of the association between Int7G24A polymorphism and cancer risk applying dominant model. The triangles and horizontal lines correspond to the subgroup-specific ORs and 95% CIs. The vertical solid line shows the OR of 1. Especially, “Other” indicates other kind of cancer in addition to breast cancer and colorectal cancer, including 2 ALL studies (T-lineage and B-lineage), 1 cervix cancer study, 1 NSCLC study, 1 osteosarcoma study, 1 RCC study, 1 TCC study, 1 ESCC study, 1 colon adenoma study, and 1 GCA study; “Mixed” means mixed population with Caucasian and African-American.

Results of subgroup analyses had been shown in Figs 5 and 6. To assess the potential effects of specific study characteristics on the association between Int7G24A polymorphism and cancer risk, we pooled the ORs and 95% CIs from the subgroups of ethnicity, control source, genotyping method, type of sample, type of cancer and sample size. When stratified by ethnicity, significant association between Int7G24A rs334354 polymorphism and cancer risk was detected among the Asian population and the mixed population in both of the allele model (Asian population, OR = 1.27, 95% CI 1.11-1.45, *P* < 0.001; mixed population, OR = 2.02, 95% CI 1.52-2.68, *P* < 0.001) and dominant model (Asian population, OR = 1.28, 95% CI 1.11-1.49, *P* < 0.001; mixed population, OR = 2.27, 95% CI 1.64-3.15, *P* < 0.001), while non-significant relationship was detected for the Caucasian population (allele model, OR = 1.08, 95% CI 0.92-1.26, *P* = 0.352; dominant model, OR = 1.05, 95% CI 0.87-1.26, *P* = 0.639). Notably, no heterogeneity was detected in this subgroup analysis. When stratified by control source, Int7G24A rs334354 had been found to have an increased risk for cancer risk for population-based (PB) group (OR = 1.19, 95% CI 1.04-1.35, *P* = 0.01) and hospital-based (HB) group (OR = 1.32, 95% CI = 1.06-1.63, *P* = 0.012) in allele model. Heterogeneity was confirmed in HB group with *P* < 0.001. Applying dominant model, we found significant relationship only in PB group (OR = 1.22, 95% CI 1.07-1.39, *P* = 0.003) but not in HB group (OR = 1.29, 95% CI 0.99-1.68, *P* = 0.057), with heterogeneity found in HB group (*P*_h_ < 0.001). According to the type of sample for genotyping, significantly increased relationship was found using allele model (blood sample group, OR = 1.13, 95% CI 1.00-1.27, *P* = 0.031; tissue sample group, OR = 1.62, 95% CI 1.27-2.07, *P* = 0.150), while only the tissue sample group was detected to increase the risk for cancer (blood sample group, OR = 1.11, 95% CI 0.96-1.29, *P* = 0.028; tissue sample group, OR = 1.68, 95% CI 1.21-2.34,*P* = 0.086). Subgroup analysis was conducted among 3 methods for genotyping. In the PCR-RFLP group and PCR- SSCP group, significantly increased associations between Int7G24A rs334354 polymorphism and cancer risk were found in allele model (PCR-RFLP group, OR = 1.19. 95% CI 1.05-1.35, *P* = 0.005; PCR- SSCP group, OR = 1.81, 95% CI 1.37-2.38, *P* < 0.001) and dominant model (PCR-RFLP group, OR = 1.19. 95% CI 1.04-1.36, *P* = 0.011; PCR- SSCP group, OR = 1.88, 95% CI 1.27-2.79, *P* < 0.002), with no heterogeneity confirmed. In contrast, no relationship was detected in TaqMan group applying allele model (OR = 1.07, 95% CI 0.81-1.41, *P* = 0.649, *P*_h_ = 0.011) and dominant model (OR = 1.03, 95% CI 0.73-1.46, *P* = 0.865, *P*_h_ = 0.006). In this subgroup analysis, the studies on cancer type were further studied. However, no significant association was found between Int7G24A rs334354 and colorectal cancer in the two models (allele model, OR = 1.08, 95% CI 0.88-1.33, *P* = 0.469; dominant model OR = 1.03, 95% CI 0.78-1.36, *P* = 0.834). As for the breast cancer group, significant association was only found in allele model (OR = 1.34, 95% CI 1.15-1.56, *P* = 0.314) but not dominant model (OR = 1.53, 95% CI 0.65-3.59, *P* = 0.326). Among the group of other type cancer, Int7G24A polymorphism was confirmed to increase the risk for cancer (allele model, OR = 1.25, 95% CI 1.09-1.42, *P* < 0.001; dominant model, OR = 1.34, 95% CI 1.14-1.57, *P* < 0.001). Moreover, in order to explore the confounding effect of sample size on the studying association, we also conducted the subgroup analysis according to sample size. As for the group with sample size larger than 1000, significantly increased association was found in both allele model (OR = 1.18, 95% CI 1.06-1.31, *P* = 0.002) and dominant model (OR = 1.18, 95% CI 1.06-1.32, *P* = 0.003). In contrast, no association was detected among the group with sample size smaller than 300 (allele model, OR = 1.34, 95% CI 0.75-2.40, *P* = 0.322; dominant model, OR = 1.35, 95% CI 0.64-2.84, *P* = 0.431). Among the group with sample size larger than 300 (including 300) and smaller than 1000 (including 1000), increased relationship was only found in allele model (OR = 1.63, 95% CI 1.10-2.42, *P* = 0.016) but not dominant model (OR = 1.59, 95% CI 0.92-2.77, *P* = 0.100). Detailed results were shown in Figs 5 and 6.

### Sensitivity analyses

**Figure 7:**
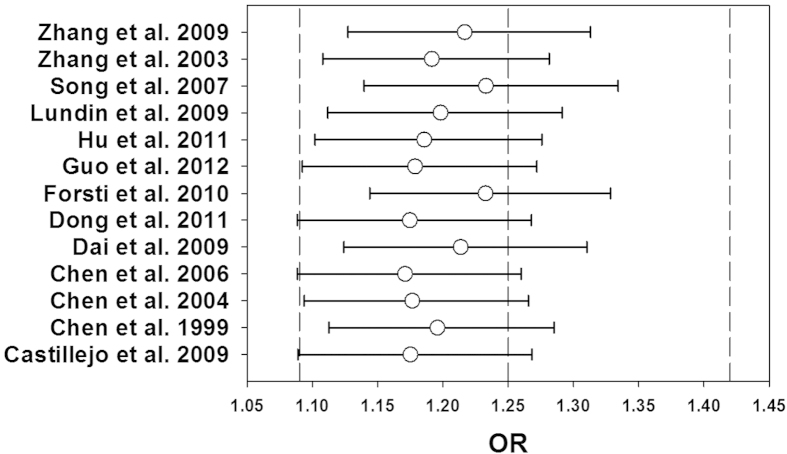
Results of leave-one-out method using allele model. The circles and the horizontal lines represent the ORs and 95% CIs after omitting studies in turn. The vertical dashed lines show the ORs of 1.09, 1.25 and 1.42.

**Figure 8:**
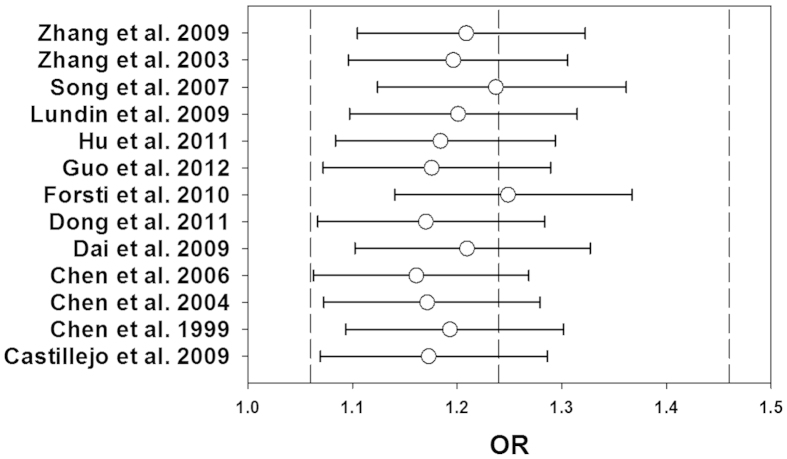
Results of leave-one-out method using dominant model. The circles and the horizontal lines represent the ORs and 95% CIs after omitting studies in turn. The vertical dashed lines show the ORs of 1.06, 1.24 and 1.46.

Sensitivity analyses were used to evaluate the sensitivity of this meta-analysis. Firstly, fixed-effect model and quality-effect model were compared with random-effect models, and the conclusions remained unchanged in allele model (see Fig. 5, random-effect model, OR = 1.25, 95% CI = 1.09-1.42, *P* = 0.001; fixed-effect model, OR = 1.20, 95% CI = 1.12-1.29, *P* < 0.001; quality-effect model, OR = 1.57, 95% CI = 1.06-1.46, *P* < 0.001) and dominant model (see Fig. 6, random-effect model, OR = 1.24, 95% CI = 1.06-1.46, *P* = 0.001; fixed-effect model, OR = 1.20, 95%CI = 1.10-1.30, *P* < 0.001; quality-effect model, OR = 1.19, 95% CI = 1.01-1.41, *P < *0.001). Secondly, we applied leave-one-out method by excluding one study in turn to evaluate the stability of the obtained conclusions. The results of the leave-one-out method had been shown in Figs 7 and 8.The statistical significance of the results was not altered when any single study was omitted, confirming the stability of the results. Especially, the genotype distributions of control groups in 1 study^24^ did not follow HWE, but the corresponding pooled ORs was not significantly altered by the exclusion of this study. Therefore, the results of this meta-analysis were stable and robust.

### Publication bias

Publication bias was assessed through the visual inspection of funnel plots and with tests of Begg rank correlation and Egger regression asymmetry. The shape of funnel plots did not reveal any evidence of obvious asymmetry in all comparisons in overall population. In addition, the Egger test (*P* = 0.464 for allele model, *P* = 0.287 for dominant model) and Begg test (*P* = 0.669 for allele model, *P* = 0.502 for dominant model) did not suggest evidence of publication bias at a significant level of *P* = 0.05.

### Power calculation

Based on the data in HapMap database (http://www.hapmap.org), G allele distributions of Int7G24A variant were 78% for Utah residents with ancestry from Northern and Western Europe (CEU) and 51% for Han Chinese in Beijing (CHB) and A allele distributions were 22% for CEU and 49% for CHB. In our meta-analysis, the minor allele frequency (MAF) of Int7G24A variant was set to be 0.22. Power analyses were performed on the basis of the least effect size suggested in our meta-analysis (dominant model, OR = 1.24) under the assumption for the alpha value of 0.01 and the number of case-control pairs of 4092. When the allele frequency of A allele was set to be 0.49, our meta-analysis had a power of 98.6% to detect an OR of 1.24 for the association between Int7G24A polymorphism and cancer risk. When the allele frequency of A was set to be 0.22, our meta-analysis had a power of 99.7% to detect an OR of 1.24 for the association between Int7G24A polymorphism and cancer risk.

## Discussion

Evidence of epidemiology studies, mechanism researches and animal experiments had confirmed the important role for genetic polymorphism in the development and progression of cancer, especially for the genes involved in tumorigenesis^27^,^28^. Although a multitude of novel genetic factors that may contribute to the susceptibility of cancer have been identified by the genome-wide association studies (GWAS) and epidemiological studies in the past few years, there is still a great need to further explore other genetic factors which may lead to the susceptibility of cancer but with low-penetrance effect. The transforming growth factor β (TGF-β) signaling pathway is a key player in metazoan biology, and its misregulation can result in tumor development^29^,^30^. On one side, in normal and premalignant cells, TGF-β enforces homeostasis and suppresses tumor progression directly through cell-autonomous tumor-suppressive effects (cytostasis, differentiation, apoptosis) or indirectly through effects on the stroma (suppression of inflammation and stroma-derived mitogens). On the other side, when cancer cells lose TGF-β tumor-suppressive responses, they can use TGF-β to their advantage to initiate immune evasion, growth factor production, differentiation into an invasive phenotype, and metastatic dissemination or to establish and expand metastatic colonies^4^,^6^. As an indispensable member of the TGF-β family, several mutations in the gene had been found to be related with cancer risk, including polymorphism of Int7G24A rs334354^14^,^31^,^32^,^33^. Since the identification of the potential association between Int7G24A (rs334354) polymorphism and cancer risk^18^, an increasing number of relevant studies had been conducted with results suggesting the important role of Int7G24A rs334354 mutation in cancer development^12^,^13^,^19^. However, the conclusions from these studies were inconsistent and controversial, primarily resulting from the insufficient sample size to give the right answer. In our meta-analysis, after combing all relevant studies, 13 studies including 4092 cases and 5909 controls were studied. Power analysis indicated that we had a power of 98.6% under the allele frequency of 0.49 and 99.7% under the allele frequency of 0.22 to detect an OR of 1.24 for the association between Int7G24A polymorphism and cancer risk, based on the sample size of our analysis. The results of the overall population had indicated that Int7G24A rs334354 polymorphism had an increased risk for cancer development, reaching significant levels at both of the 2 genetic models. With respect to subgroup analysis, the association still remained significant in many groups.

Traditionally, most meta-analyses on gene-disease studies would test multiple genetic models, which did not estimate the magnitude of effect of a molecular association, leading to improper and confused conclusions. Thus, in our meta-analysis, a method to choose the best genetic model for case-control genetic association studies was applied. Several methods^34^,^35^,^36^ have been considered and the method developed by Bagos *et al*.^36^ was determined. The method, making use of the binary structure of the data, and by treating the genotypes as independent variables in a logistic regression, was a simple and commonly used methodology that performs satisfactorily and flexibly. This methodology was reported to avoid multiple comparisons, and directly tested the most probable model of genetic inheritance in meta-analyses of gene–disease association studies. In our analysis, dominant model was indicated to be the best genetic model for clarifying the association between Int7G24A polymorphism and cancer risk. Besides, allele model was conducted to explore the difference between A carriers and G carriers on the risk for cancer. Thus, allele model and dominant model were applied in our meta-analysis.

Heterogeneity between studies is common in the meta-analysis of genetic association studies. In the present meta-analysis, heterogeneity was determined by Q-test and statistically significant heterogeneity was confirmed within allele model (*P*_h_ = 0.001) and dominant model (*P*_h_ < 0.001). To explore the potential heterogeneity among studies, subgroup analyses were conducted in our meta-analysis. In the subgroup analysis by ethnicity, the heterogeneity was effectively removed, suggesting that the present heterogeneity was partly derived from ethnicity. It is well known that genotype distributions vary between populations, and genetic studies on the genotype-disease association are generally performed on specific population. With respect to Int7G24A rs334354, the genotype frequencies also differ between ethnicities (G allele: 78% for CEU, 51% for CHB; A allele: 22% for CEU, 49% for CHB). Thus, conclusions of genetic studies performed in various countries might be different. In our work, significant association was found among Asian population (allele model, OR = 1.27, 95% CI 1.11-1.45, *P* < 0.001; dominant model, OR = 1.28, 95% CI 1.11-1.49, *P* < 0.001), whereas there was non-significant relationship was detected in the two genetic models among Caucasian population (allele model, OR = 1.08, 95% CI 0.92-1.26, *P* = 0.352; dominant model, OR = 1.05, 95% CI 0.87-1.26, *P* = 0.639). This result had indicated that the carcinogenesis of Int7G24A polymorphism might be effective only among Asian population but not Caucasian population. Interestingly, it was noted that when stratified by control population, heterogeneity appeared only in the hospital-based (HB) group but not the population-based (PB) group. Besides, in HB group (allele model, OR = 1.32, 95% CI = 1.06-1.63, *P* = 0.012; dominant model, OR = 1.29, 95% CI 0.99-1.68, *P* = 0.057), there was a great risk to develop cancer compared with PB group (allele model, OR = 1.19, 95% CI 1.04-1.35, *P* = 0.01; OR = 1.22, 95% CI 1.07-1.39, *P* = 0.003). Selection bias might be the reason of this result. On one side, since the HB group were hospital patients, they could not validly represent the exposure situation of the overall population. On the other side, mostly, the HB controls were not randomly selected from the whole patient population. Thus, results from the population-based controls were thought to be more reliable.

In addition, analysis by cancer type was conducted for further study. Present meta-analysis included 4 studies of colorectal cancer, 2 studies of breast cancer and 7 studies of other type of cancer. As for colorectal cancer, no significant association with Int7G24A rs334354 polymorphism was found in both allele model and dominant model (allele model, OR = 1.08, 95% CI = 0.88-1.33; dominant model, OR = 1.03, 95% CI = 0.78-1.36), compared with the previous meta-analysis including 3 studies by Zhang in 2012^16^ (heterozygote model, OR = 0.97, 95% CI = 0.67-1.42; homozygote model, OR = 1.68, 95% CI = 1.14-2.47; recessive model, pooled OR = 1.71, 95% CI = 1.17-2.51). In Zhang’s study, colon adenoma cases were misclassified into colorectal cancer group^23^. Strictly speaking, colon adenoma should be labeled as the precancerous lesion of colorectal cancer more than one kind of it. Thus, heterogeneity will occur when combining colon adenoma cases with colorectal cancer cases. In our meta-analysis, colon adenoma cases were excluded since the outcome of interest is cancer. However, false positive might appear resulting from the lack of relevant studies and the small sample size. More relevant studies were needed to gain a more reliable.

Some limitations should be considered for our meta-analysis. Firstly, potential biases were hardly inevitable in our analysis. Our restriction on searching studies published in indexed journals could bring in biases such as time-lag bias and publication bias, though the publication bias was not found in the present meta-analysis. Besides, non-differential misclassification bias was possible because the included controls were not uniform. These controls were likely to develop cancer in subsequent years though no clinical symptoms was observed at the time of investigation. Moreover, lack of information for the adjustments of major confounders including age, smoking status, drinking status and environmental factors might cause confounding bias. Secondly, the number of included studies for Int7G24A rs334354 polymorphism was limited for further analysis due to shortage of original studies. More larger and well-designed studies were needed to confirm our results. Thirdly, there are only three ethnicity groups (Asian, Caucasian, mixed) included in the present paper. Thus, it was doubtful whether the obtained conclusions were generalizable to other population.

In conclusion, the present meta-analysis of 13 studies including 4092 cases and 5909 controls suggested that Int7G24A rs334354 polymorphism of gene TGFBR1 involved in the TGF-β signaling pathway had a significantly increased risk on the risk for cancer in both of the two genetic models. Additionally, compared with Caucasian population, Asian population with Int7G24A polymorphism had been found to have a greater risk for the development of cancer. However, well-designed studies with larger sample size and more ethnic groups are required to validate the risk identified in the current meta-analysis.

## Methods

### Search strategy

In this paper, we conducted a literature search on PubMed (Medline), Embase, Web of Science and Chinese National Knowledge Infrastructure (CNKI) from January 1966 through August 2014 for case-control studies examining the association between Int7G24A (rs334354) polymorphism of TGFBR1 and the risk for cancer, applying the search terms “TGFBR1 or transforming growth factor receptor 1 or type 1 TGF-beta receptor”, “polymorphism or mutation or variation or genotype or SNP” and “cancer or tumor or neoplasm or carcinoma”. Besides, reference lists from retrieved articles were also reviewed. Only articles published in the English and Chinese were considered. We conducted our meta-analysis according to the PRISMA checklists and followed the guideline^37^.

### Inclusion criteria

Studies meeting the following criteria were included in the meta-analysis: (1) the study design was case-control (2) the outcome of interest was cancer, (3) the study evaluated the association between Int7G24A rs334354 polymorphism and the risk for cancer, (4) the study reported sufficient data to calculate odds ratios (ORs) and their 95% confidence intervals (CI), (5) the study should be human research. Additionally, we excluded reviews, editorials, non-human studies, and letters without sufficient data. When multiple reports based on the same study were published, only the most recent or complete report could be used.

### Data extraction

Eligibility evaluation and data extraction were carried out independently by two reviewers (W. W. and Y.T.). Discrepancies were adjudicated by discussion with a third reviewer (Q.L.). The following information was extracted from all the identified studies: name of first author, year of publication, country where the study was performed, ethnicity, type of control source, type of cancer, method for genotyping, total number of cases and controls, and the frequencies of every genotype.

### Quality assessment

The qualities of the included studies were accessed by two authors respectively according to a set of predetermined criteria (Table 2), which was extracted and modified from previous studies^38^. In this scale for quality assessment, six items, including the representativeness of cases, source of controls, ascertainment of cancer, quality control of genotyping methods, sample size and HWE, were carefully checked. P value less than 0.01 was considered significant departure from HWE. The total scores ranged from 0 to 10, with higher scores indicating better quality. Two investigators scored the studies independently and solved disagreement through discussions.

### Statistical analyse**s**

The heterogeneity among studies was estimated by the Cochran Q test, which confirmed the heterogeneity at a significance level of *P* ≤ 0.10. Fisher’s exact test was used to check for deviations from Hardy–Weinberg equilibrium (HWE) among the controls in each study^39^. A method for meta-analysis of case-control genetic association studies using logistic regression developed by Bagos *et al*.^36^ was conducted in our paper. This method, making use of the binary structure of the data, and by treating the genotypes as independent variables in a logistic regression, was a simple and commonly used methodology that performs satisfactorily and flexibly. Considering A is the allele reported to be related with increased risk for disease, parameters *θ*_2_ and *θ*_3_ are log OR_GA/GG_ and log OR _AA/GG_, respectively, as defined by the following logistic regression model:




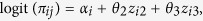




where *π*_*ij*_ is the disease risk for the *j*th genotype in the *i*th study, and z_*i2*_ and z_*i3*_ are dummy variables indicating genotypes GA and AA, respectively. If there was heterogeneity on at least one of these odds ratios (OR_GA/GG_ and OR _AA/GG)_, random-effect model^36^ would be used to pool the effect, whereas fixed-effect model would be applied. The appropriate genetic model was determined based on the following relationship between *θ*_2_ and *θ*_3_, as follow:

1. No association: *θ*_2_ = *θ*_3_ = 0 (OR_GA/GG_ = OR_AA/GG_ = 1);
2. Recessive model: *θ*_2_ = 0 (OR_GA/GG_ = 1) and *θ*_3 _≠ 0 (OR_AA/GG _≠ 1);
3. Dominant model: *θ*_2 _≠ 0, *θ*_3 _≠ 0 and *θ*_2_ = *θ*_3_ (OR_GA/GG_ = OR_AA/GG _≠ 1);
4. Multiplicative codominant model: *θ*_2 _≠ 0, *θ*_3 _≠ 0 and 2*θ*_2_ = *θ*_3_ (OR^2^_GA/GG_ = OR_AA/GG_).

Once the best genetic model was identified, the three genotypes were collapsed into two groups to obtain the pooled results. Notably, Among the 13 studies, there were 2 studies^13^,^18^ which had cells with no count. Considering the potential risk of inflating the magnitude of the pooled effect after the exclusion of studies with zero cell counts, these studies will be included in our meta-analysis^40^. A common way to deal with this problem is to add 0.5 to each cell of the 2 × 2 table for the study^41^. In our work, this correction to all cell counts was automatically added by STATA^42^. If there were more than one type of cancer reported in one study, total number of participants reported to be cancer case would be extracted and compared them with the control group.

To explore the potential heterogeneity among studies, subgroup analyses were conducted according to ethnicity, control source, genotyping method, type of sample, type of cancer and sample size. In addition, sensitivity analyses were employed to find potential origins of heterogeneity and to examine the influence of various exclusions on the combined OR. Besides, the results from quality-effect model was introduced to be compared with those from random-effect model and fixed-effect model^43^ to evaluate the sensitivity of our results. Publication bias was evaluated through the visual inspection of funnel plots and with tests of Begg rank correlation^44^ and Egger regression asymmetry^45^. *P* < 0.05 was considered to be representative of a significant statistical publication bias. Forest plots were applied to assess the association between Int7G24A rs334354 polymorphism and cancer risk. Power analysis was performed to calculate the power for our meta-analysis to detect the estimated risk of the association between Int7G24A polymorphism and cancer risk. In addition to the power calculation (using Quanto version 1.2.4) and quality-effect modeling (applying MetaXL version 2.2 software), all the other statistical analyses were performed with STATA version 12.0 software (Stata Corporation, College Station, Texas, United States). All reported probabilities (*P* values) were two-sided.

## Additional Information

**How to cite this article**: Wu, W. *et al*. Association between Int7G24A rs334354 polymorphism and cancer risk: a meta-analysis of case-control studies. *Sci. Rep*. **5**, 11350; doi: 10.1038/srep11350 (2015).

## Figures and Tables

**Table 1:** Characteristics of included studies on association between Int7G24A rs334354 polymorphism and cancer risk.

Study	Country	Ethnicity	Control Source	Cancer Type	Genotyping Method	Sample Size (case/control)	Genotype Distribution (case/control)					HWE	QA
							GG	GA	AA	G-allele	A-allele		
Dai *et al*. 2009	German	Caucasian	PB	ALL	TaqMan	538/551	307/356	176/170	25/25	850/882	226/220	Yes	7
Zhang *et al*. 2009	China	Asian	PB	Colorectal	PCR-RFLP	206/838	60/245	103/431	43/162	223/921	189/755	Yes	9
Song *et al*. 2007	Sweden	Caucasian	HB	Breast	PCR-RFLP	767/853	500/559	238/265	29/29	1238/1383	296/323	Yes	6
Zhang *et al*. 2003	China	Asian	HB	NSCLC	PCR-SSCP	53/89	18/31	24/52	11/6	60/114	46/64	No	6
Lundin *et al*. 2009	Sweden	Caucasian	HB	Colorectal	PCR-RFLP	214/853	135/559	67/265	12/29	337/1383	91/323	Yes	8
Hu *et al*. 2011	China	Asian	HB	Osteosarcoma	PCR-RFLP	168/168	100/115	57/48	11/5	257/278	79/58	Yes	6
Guo *et al*. 2012	China	Asian	PB	GCA	PCR-RFLP	468/584	291/402	155/168	22/14	737/972	199/196	Yes	7
Forsti *et al*. 2010	Sweden	Caucasian	HB	Colorectal	TaqMan	302/581	220/382	68/179	14/20	508/943	96/219	Yes	9
Chen *et al*. 2004	USA	Mixed	HB	RCC and TCC	PCR-SSCP	151/113	79/81	64/32	8/0	222/194	80/32	Yes	6
Chen *et al*. 2006	USA	Mixed	HB	Breast	PCR-SSCP	223/153	120/113	92/37	11/3	332/263	114/43	Yes	7
Castillejo *et al*. 2009	Spain	Caucasian	HB	Colorectal	TaqMan	504/504	296/333	178/156	30/15	770/822	238/186	Yes	6
Dong *et al*. 2011	China	Asian	PB	ESCC	PCR-RFLP	482/584	296/402	163/168	23/14	755/972	209/196	Yes	9
Chen *et al*. 1999	Netherland	Mixed	PB	Cervix	PCR-SSCP	16/38	9/24	7/12	0/2	25/60	7/16	Yes	3

Mixed, mixed population with Caucasian and African-American; PB, population-based control; HB, hospital-based control; ALL, acute lymphoblastic leukemia; NSCLC, non-small cell lung cancer; GCA, gastric cardia adenocarcinoma; RCC, renal cell carcinoma; TCC, transitional cell carcinoma of upper urinary tract and bladder; ESCC, esophageal squamous cell carcinoma; QA, quality assessment using the Newcastle-Ottawa Scale for case-control studies.

**Table 2:** Scale for quality assessment.

Criteria	Score
1. Representativeness of cases	
Consecutive/randomly selected from case population with clearly defined sampling frame	2
Consecutive/randomly selected from case population without clearly defined sampling frame or with extensive inclusion/exclusion criteria	1
Not Mentioned	0
2. Source of controls	
Controls were consecutive/randomly drawn from the same sampling frame (ward/community) as cases	2
Controls were consecutive/randomly drawn from a different sampling frame as cases	1
Not described	0
3. Ascertainment of cancer	
Histological confirmation at the Department of Pathology	2
Patient medical record	1
Not described	0
3. Quality control of genotyping methods	
Genotyping done under “blinded” condition	1
Unblinded or not mentioned	0
4. Sample size	
>1000	2
300–1000	1
<300	0
5. Hardy-Weinberg equilibrium (HWE)	
Hardy-Weinberg equilibrium in control subjects	1
Hardy-Weinberg disequilibrium in control subjects	0
